# *In vivo* analysis of noise dependent activation of white blood cells and microvascular dysfunction in mice

**DOI:** 10.1016/j.mex.2021.101540

**Published:** 2021-10-08

**Authors:** Jonas Eckrich, Yue Ruan, Subao Jiang, Katie Frenis, Giovanny Rodriguez-Blanco, Alexander Philippe Maas, Maria Teresa Bayo Jimenez, Marin Kuntic, Matthias Oelze, Omar Hahad, Huige Li, Sebastian Steven, Sebastian Strieth, Alex von Kriegsheim, Thomas Münzel, Andreas Daiber, Adrian Gericke, Benjamin Philipp Ernst

**Affiliations:** a Department of Otorhinolaryngology, University Medical Center Bonn (UKB), Bonn, Germany; b Department of Ophthalmology, University Medical Center of the Johannes Gutenberg University Mainz, Germany; c Department of Cardiology, Cardiology I, University Medical Center of the Johannes Gutenberg University Mainz, Mainz, Germany; d Institute of Genetics and Cancer, University of Edinburgh, United Kingdom; e German Center for Cardiovascular Research (DZHK), Partner Site Rhine-Main, Mainz, Germany; f Department of Pharmacology, University Medical Center of the Johannes Gutenberg University Mainz, Germany

**Keywords:** Dorsal skinfold chamber, Fluorescent labeling of blood cells, Cerebral arterioles cannulation, Video microscopy

## Abstract

This article contains supporting information on data collection for the research article entitled “Aircraft noise exposure drives the activation of white blood cells and induces microvascular dysfunction in mice” by Eckrich et al. We found that noise-induced stress triggered microvascular dysfunction via involvement of innate immune-derived reactive oxygen species. In this article, we present the instrumentation of mice with dorsal skinfold chambers for *in vivo* microscopic imaging of blood flow, interaction of leukocytes with the vascular wall (also by fluorescent labelling of blood cells) and vessel diameter. In addition, we explain the preparation of cerebral arterioles for measurement of vascular reactivity *in vitro*.

• visualization of noise-dependent effects in dorsal skinfold chamber.
• *in vivo* microscopy of noise-dependent activation of white blood cells.
• analysis of noise-dependent microvascular dysfunction in dorsal skinfold chamber and cannulated cerebral arterioles.

## Graphical abstract

**Figure:**
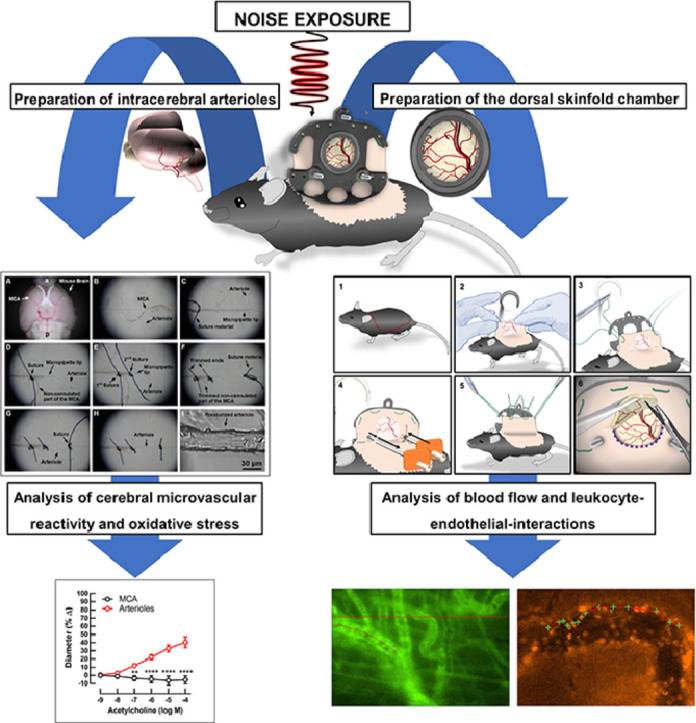

Specifications table

**Table:** 

Subject Area:	Medicine and Dentistry
More specific subject area:	Microvascular analysis of noise induced inflammation and microvascular dysfunction
Method name:	*In vivo* fluorescence microscopy and cerebral arteriole cannulation to assess noise induced changes in activation of white blood cells and microvascular dysfunction
Name and reference of original method:	*In vivo* microscopy of dorsal skinfold chambers • *Algire GH, Legallais FY. Recent developments in the transparent-chamber technique as adapted to the mouse. J Natl Cancer Inst. 1949;10(2):225-53, incl 8 pl. Epub 1949/10/01. PubMed PMID:**15393709**.* • *Sandison JC. A new method for the microscopic study of living growing tissues by the introduction of a transparent chamber in the rabbit's ear. The Anatomical Record. 1924;28(4):281-7. doi: https://doi.org/10.1002/ar.1090280403*. • *Endrich B, Asaishi K, Gotz A, Messmer K. Technical report–a new chamber technique for microvascular studies in unanesthetized hamsters. Res Exp Med (Berl). 1980;177(2):125-34. Epub 1980/01/01. doi: 10.1007/BF01851841. PubMed PMID: 7003665.* *Cerebral arteriole cannulation* • *Reference of original method is not available. It was first described by our working group:* *Eckrich J, Frenis K, Rodriguez-Blanco G, Ruan Y, Jiang S, Bayo Jimenez MT, et al. Aircraft noise exposure drives the activation of white blood cells and induces microvascular dysfunction in mice. Redox Biol. 2021;46:102063. Epub 2021/07/19. doi: 10.1016/j.redox.2021.102063. PubMed PMID:**34274810**.*
Resource availability:	*Dorsal skinfold chamber preparation* *Titanium frame of dorsal skinfold chamber (custom made)* *Autoclave (Tuttnauer systec 5050 ELVD, Tuttnauer Europe B.V., Breda, Netherlands)* *Ketamine (Ketanest; Pfizer Pharma GmbH, Berlin, Germany)* *Xylazine (Rompun; Bayer, Leverkusen, Germany)* *NaCl 0.9% (B.Braun 0,9% Mini-Plasco® connect, B. Braun Melsungen AG, Melsungen, Germany)* *Heated operation platform (MEDAX GmbH & Co KG, Neumünster, Germany)* *Eye ointment (Corneregel®, Bausch & Lomb Inc., Rochester, New York, USA; Bepathen® Eye and Nose Ointment, Bayer AG, Leverkusen, Germany)* *Electric razor (Remington Contour, Spectrum Brands, Middleton, Wisconsin, USA)* *Depilatory cream (Veet, Reckitt Benckiser Deutschland GmbH, Heidelberg, Germany)* *Disinfectant spray (octenisept spray, Schülke & Mayr GmbH, Norderstedt, Germany)* *Surgical sutures (ETHIBOND EXCEL V5 - USP 4-0, 0.75m, Ethicon Inc., Raritan, New Jersey, USA)* *1,5 mm Strauss cannula (DISPOMED GmbH & Co. KG, Gelnhausen, Germany)* *surgical pen (Securline Skin Marker, Aspen Surgical Products, Caledonia, Michigan, USA).* *Surgical microscope (ZEISS OPMI 1 FC, Carl Zeiss Meditec AG, Oberkochen, Germany)* *Cover glass slip (11.8 mm, Hecht Assistent, Glaswarenfabrik Karl Hecht GmbH & Co KG, Sondheim, Germany)* *Analgesia (Tramadol-ratiopharm, ratiopharm GmbH, Ulm, Germany)* *In vivo microscopy* *Fluorescein isothiocyanate (FITC)-labelled dextran (average mol wt 500 000, Sigma, Deisenhofen, Germany)* *Rhodamine 6G (Sigma, Deisenhofen, Germany)* *Local anesthesia (Emla® 25 mg/g Lidocain +25 mg/g Prilocain creme, Aspen Germany GmbH, Munich, Germany)*
	*Surgical canulla( 0.3 × 13mm, BD Microlance 3, Becton, Dickinson and Company Ltd., Drogheda, Ireland)* *Intravital microscope (Olympus BXFM, Olympus Deutschland GmbH, Hamburg, Germany)* *Cell Sens Dimension (Olympus Deutschland GmbH, Hamburg, Germany)* *Off-line computer-aided videoframe analysis (Cap Image, Dr. Zeintl Ingenieurbüro, Dreieich, Germany)* *Mechanical hand counter (Roth Selection, Carl Roth GmbH + Co. KG, Karlsruhe, Germany)* *Cerebral arteriole studies* *Precision tweezers (type 5, straight with extra fine tips, LH53.1, Carl Roth GmbH + Co. KG, Karlsruhe, Germany)* *Vannas capsulotomy scissors (straight, G-19760, Geuder AG, Heidelberg, Germany)* *Glass Capillaries (1.2 × 0.8 mm outer/inner diameter, 9-000-1211, Drummond Scientific Company, Broomall, PA, USA)* *Nylon Suture (10-0, 198001, Alcon AG, Freiburg, Switzerland)* *Vertical Pipette Puller (Model 700C, David Kopf Instruments, Tujunga, CA, USA)* *Pressure Myograph (Jim's Instruments Manufacturing Inc., Iowa City, IA, USA)* *Pericyclic pump (Model CYCLO II, EP76.1, Carl Roth GmbH + Co. KG, Karlsruhe, Germany)* *Recirculating water bath (Model FE 2, Thermo Haake GmbH, Karlsruhe, Germany)* *Inverted microscope (Model SXB-1A, Lowest Price Microscopes, MO, USA)* *Digital camera (Model TK-C1381, JVC Deutschland GmbH, Bad Vilbel, Germany)* *Calcium chloride (5239.1, Carl Roth GmbH + Co. KG, Karlsruhe, Germany)* *Kalium chloride (6781.1, Carl Roth GmbH + Co. KG, Karlsruhe, Germany)* *Kalium dihydrogen phosphate (3904.2, Carl Roth GmbH + Co. KG, Karlsruhe, Germany)* *Magnesium sulphate (261.2, Carl Roth GmbH + Co. KG, Karlsruhe, Germany)* *Sodium chloride (9265.2, Carl Roth GmbH + Co. KG, Karlsruhe, Germany)* *Sodium hydrogen carbonate (0965.3, Carl Roth GmbH + Co. KG, Karlsruhe, Germany)* *α-(D)-(+)- Glucose monohydrate (6780.1, Carl Roth GmbH + Co. KG, Karlsruhe, Germany)* *9,11-dideoxy-9α,11α-methanoepoxy prostaglandin F2α (U-46619, 16450, Cayman Chemical, Ann Arbor, MI, USA)* *Acetylcholine chloride (A6625-25G, Merck KGaA, Darmstadt, Germany)* *Sodium nitroprusside (1065410025, Merck KGaA, Darmstadt, Germany)*

## Introduction

The influence of noise exposure on microvascular dysfunction, inflammation and changes in blood flow remains the subject of intense investigation at the epidemiological level in large cohorts [1] and at the mechanistic level in mouse studies [2], [3], [4], [5]. Since inflammation ultimately leads to pathophysiological alterations of blood flow and the adherence and diapedesis of leukocytes, visualization of these changes has been the main purpose of various invasive and non-invasive techniques, exposing the vascular network to scientific investigation [6,7].

The dorsal skinfold chamber facilitates an unobstructed insight to the intramuscular vessel network through exposure of the panniculus carnosus muscle in mice. Moreover, application of fluorophores allows the visualization blood plasma [8] and specific blood cells, including, leukocytes [9], and thrombocytes [10].

## Dorsal skinfold chamber preparation

For the specific research performed by our working group [11], male C57BL/6j or gp91phox^−/y^ mice, aged > 8 weeks (>25 g body weight) are used as an experimental model, however, for other mouse strains, including immune deficient mice, chamber preparation and intravital analysis has also been described with some modifications [12], [13], [14], [15], [16]. All animal experiments were approved by the Landesuntersuchungsamt Koblenz, Germany (23 177-07/G 18-1-084 and addenda). All experiments comply with the ARRIVE guidelines and were carried out in accordance with the EU Directive 2010/63/EU for animal experiments. All experimental procedures were performed according to institutional and governmental guidelines and all people involved in the experimental course were qualified to perform experimental procedures on laboratory animals (FELASA B/C accreditation). All animal experiments were approved by the Landesuntersuchungsamt Koblenz, Germany (23 177-07/G 18-1-084 and addenda).

The dorsal skinfold chambers as well as all surgical instruments have to be sterilized before any surgical intervention (Tuttnauer systec 5050 ELVD, Tuttnauer Europe B.V., Breda, Netherlands).

**Fig. 1:**
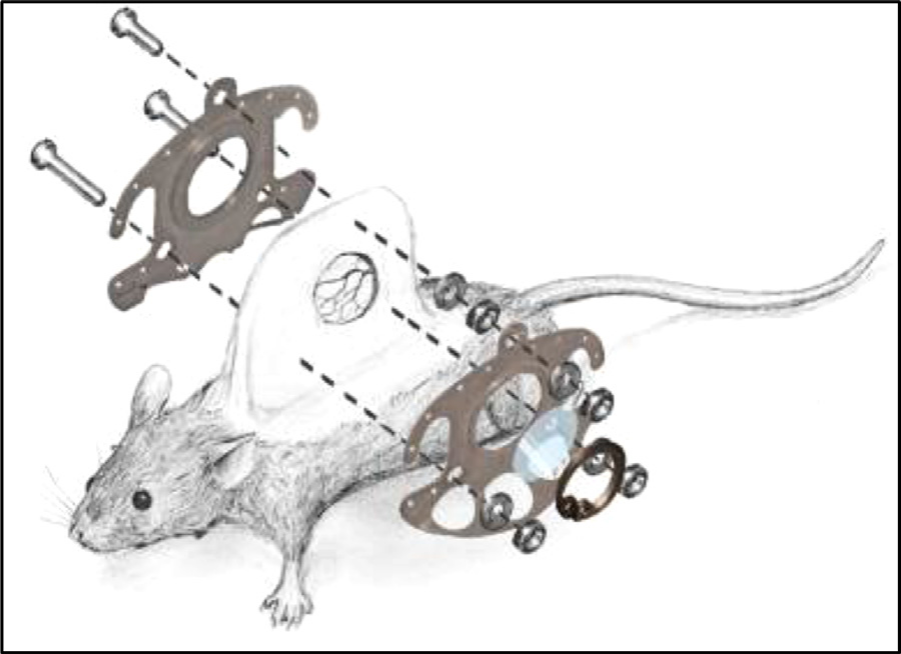
Surgical anatomy and technical equipment of the dorsal skinfold chamber in mice. Reused from [11] with permission.

The dorsal skinfold chamber (custom made) consists of two antagonizing titanium frames incorporating three canals for fixing screws as well as 12 holes for the surgical sutures (ETHIBOND EXCEL V5 - USP 4–0, 0.75m, Ethicon Inc., Raritan, New Jersey, USA) affixing the chamber to the mouse's skin (Fig. 1).

A removable cover glass is fixed with a retaining ring in order to protect the exposed tissue within the chamber window whilst allowing full visual access. The chamber implantation is performed after intraperitoneal injection of ketamine (0.1 mg/g Ketanest®; Pfizer Pharma GmbH, Berlin, Germany) and xylazine (0.01 mg/g Rompun®; Bayer, Leverkusen, Germany) mixed with NaCl 0.9% (B. Braun 0,9% Mini-Plasco® connect, B. Braun Melsungen AG, Melsungen, Germany) in a 1/10 ratio.

Since the regulation of the animals’ body temperature is significantly altered by the anesthesia, temperature homeostasis has to be carefully observed for the duration of sedation. To avoid hypothermia, animals are placed on a heated operation platform (MEDAX GmbH & Co KG, Neumünster, Germany). Furthermore, intensive eye care is of great importance. Therefore, repetitive application of eye ointment like Corneregel® (Bausch & Lomb Inc., Rochester, New York, USA) or Bepanthen® Eye and Nose Ointment (Bayer AG, Leverkusen, Germany) to avoid damage to the cornea throughout the course of anesthesia.

Before any surgical intervention, sufficient anesthesia has to be determined by a loss of positional as well as corneal and interdigital reflexes. Reevaluation of depth of anesthesia should be evaluated repetitively throughout the operative intervention. In case of insufficient sedation, 10% of the initial dose of the ketamine/xylazine solution are injected intraperitoneally every 5 min until sufficient anesthesia is achieved.

**Fig. 2:**
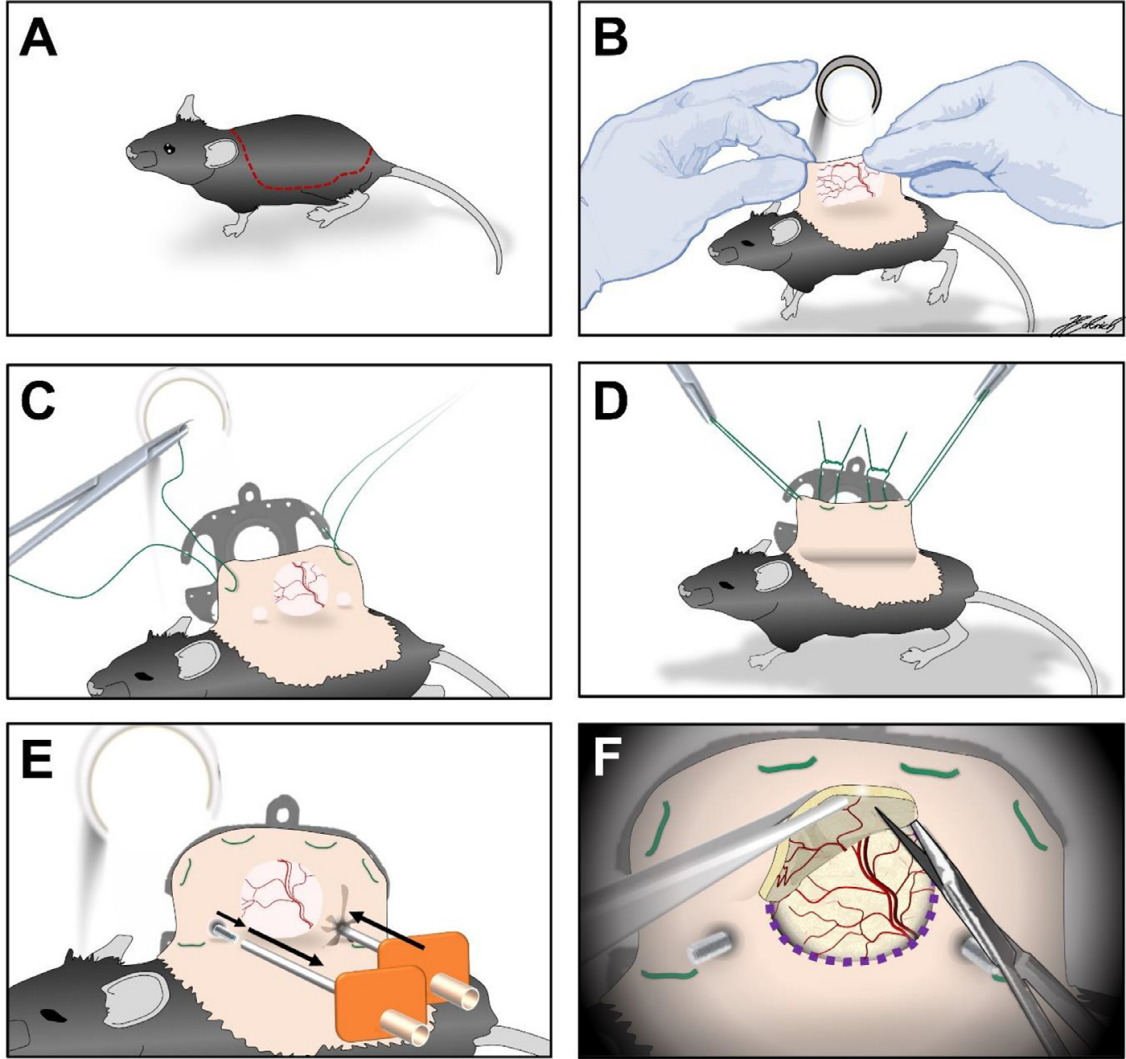
Operative procedure of the skinfold chamber preparation in detail. A: Area of depilation; B: transillumination to identify the subcutaneous vessel plexus; C: Extending the dorsal skin with sutures; D: affixing one side of the skinfold chamber with sutures. E: Anterograde piercing of the dorsal skinfold for subsequent screw insertion. F: surgical removal of skin and as well as the underlying musculocutaneous tissue with microsurgical instruments.

The chamber implantation is carried out as previously described [12]. The operation site, including the dorsal as well as the flank skin of the mouse (Fig. 2A), is first depilated with an electric razor (Remington® Contour, Spectrum Brands, Middleton, Wisconsin, USA). Razor hair removal should be carried our very carefully and a short layer of hair should be left to ensure integrity of the skin. Afterwards, depilatory cream (Veet®, Reckitt Benckiser Deutschland GmbH, Heidelberg, Germany) is applied carefully onto the dorsal skin. Three to five minutes after application the depilatory cream and the remaining hair are carefully removed with tissue wipes until the entire dorsal skin is completely depilated. Residual depilatory cream and hair is washed off with warm water (37°C). After drying, the dorsal skin is disinfected using a pre heated, alcohol free disinfectant spray (octenisept® spray, Schülke & Mayr GmbH, Norderstedt, Germany).

The dorsal skinfold is then carefully extended by hand to allow the positioning of the chamber frame in reference to the vascular network within the skins’ double layer under transillumination (Fig. 2B).

Two surgical sutures (ETHIBOND EXCEL V5 - USP 4–0, 0.75m, Ethicon Inc., Raritan, New Jersey, USA) are thread through the lateral holes in the apical part of the chamber and then retrogradely pierced through the dorsal skinfold (Fig. 2C). It is of great importance to position these sutures in a way that the chamber window is placed above a vessel branch strong in caliber in transillumination.

The two surgical threats are subsequently affixed above the chamber window, extending the dorsal skinfold. At least two further sutures should be placed at the apical and basal part of the chamber to immobilize the chamber frame on the skin (Fig. 2D).

Transillumination allows the identification of the drilling holes for the fixing screws in the chamber. The corresponding area of the skinfold is then anterogradely pierced through using a 1,5mm Strauss cannula (DISPOMED GmbH & Co. KG, Gelnhausen, Germany) (Fig. 2E). The fixing screws are subsequently inserted into the hollow cannula and retrogradely passed through the skinfold to avoid tissue damage.

By use of transillumination the detection of the external borders of the chamber window is possible, which are then marked on the dorsal skin using a surgical pen (Securline® Skin Marker, Aspen Surgical Products, Caledonia, Michigan, USA). The mouse is then positioned on its lateral side with sutured chamber frame facing down.

A surgical microscope (ZEISS OPMI® 1 FC, Carl Zeiss Meditec AG, Oberkochen, Germany) is used for the surgical removal of the skin as well as the underlying musculocutaneous tissue. With microsurgical instruments, the layer of skin facing upward as well as the apical panniculus carnosus muscle and the two layers of the retractor muscle are removed entirely within the area in correspondence to the chamber window (Fig. 2F). During the surgical removal, NaCl 0.9% (B. Braun 0,9% Mini-Plasco® connect, B. Braun Melsungen AG, Melsungen, Germany) is repetitively drizzled onto the exposed tissue to avoid drying out and consecutive tissue damage. After completion of preparation, the opposing chamber frame with an inserted cover glass slip (12 mm, Hecht Assistent, Glaswarenfabrik Karl Hecht GmbH & Co KG, Sondheim, Germany) is placed on the musculocutaneous tissue of the opposing layer within the chamber window. In a final step, the chamber is then fixed with titanium nuts and the cover glass is immobilized using a retaining ring.

In our experimental setup, the animals are treated with 0.1 mg/mL tramadol (Tramadol-ratiopharm, ratiopharm GmbH, Ulm, Germany) with the daily applied drinking water. After chamber implantation, weight and health conditions of every animal are to be monitored and documented in a score sheet quantifying indicators of an impaired wellbeing. A weight loss >20% of the postoperative bodyweight, signs of inflammation and behavioral changes indicating pain or sickness as well as signs of inflammation within the chamber window were previously determined as dropout criteria.

For an experienced operator, the entire procedure described in Fig. 2A–F will take 20–30 min per animal. In our experience, the image quality of microscopic images decreases over time due to ingrowth of connective tissue into the chamber window. This ultimately limits the capability to sufficiently monitor blood flow longer than 10–14 days, especially in small vessels.

## Noise exposure

Following chamber implantation, animals are randomized to the experimental group or control group, respectively. Animals within the experimental group are subsequently exposed to a previously-recorded playback of aircraft noise (average sound pressure level of 72 dB(A) and peak sound pressure levels of 85 dB(A), respectively) for 96 h. These sound pressure levels are roughly comparable to a passenger car passing by (72 dB(A)) or a ringing telephone/truck passing by (85 dB(A)) according to the decibel scale reported in [17]. Animals randomized to the control group are kept at homologous conditions with no noise exposure (mean background noise levels in the local animal house were approximately 48 dB(A),comparable to the noise originating from falling rain) as previously described [2,3].

## *In vivo* microscopy for analysis of microvascular reactivity and leukocyte interactions

Approximately 15 min before microscopical analysis, fluorescein isothiocyanate (FITC)-labelled dextran (Sigma, Deisenhofen, Germany; average mol wt 500 000; 0.5–0.75 mL of a 5% solution in 0.9% saline) and rhodamine 6G (Sigma, Deisenhofen, Germany, 0.5-0,75 mL of a 0.05% solution in 0.9% saline) were injected into the tail vein under local anesthesia (Emla® 25 mg/g Lidocain +25 mg/g Prilocain creme, Aspen Germany GmbH, Munich, Germany) utilizing a 0.3 × 13mm surgical canulla (BD Microlance™ 3, Becton, Dickinson and Company Ltd., Drogheda, Ireland).

FITC-labelled dextran is injected to enhance the contrast between plasma and blood cells in order to determine the specific blood flow as previously described [8].

Furthermore, rhodamine 6G is injected to visualize leukocyte-endothelial cell interactions as previously described [9].

**Fig. 3:**
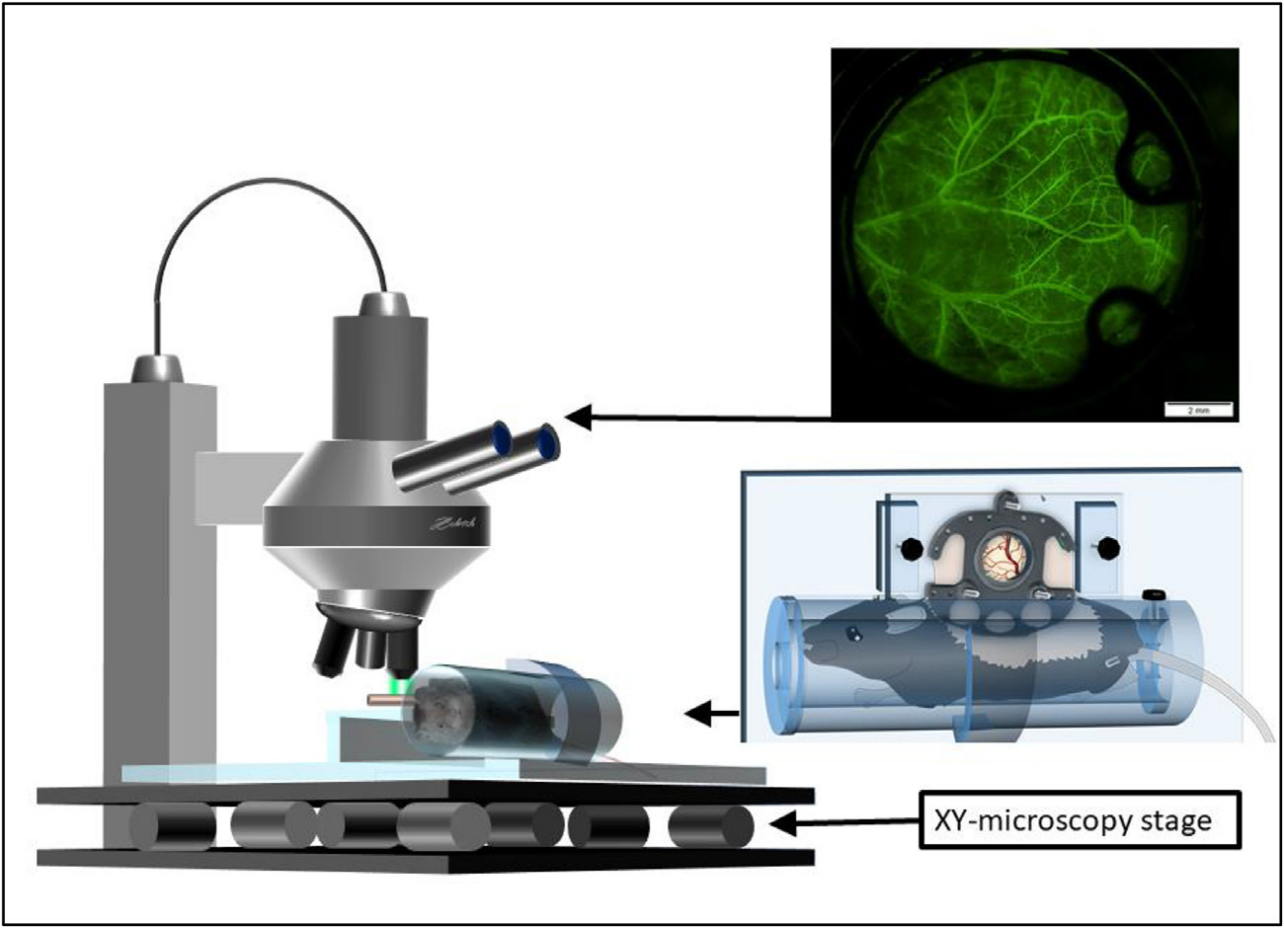
Setup of fluorescein based *in vivo* microscopy of the dorsal skinfold chamber in mice (modified). Modified from [11] with permission.

*In vivo* microscopy can be performed by immobilizing the animals in an acrylic tube, which is subsequently placed on a customized microscopy platform underneath the microscope. On this platform, the chamber is positioned and affixed horizontally to an acrylic glass frame with screws (Fig. 3). Thus, movement is sufficiently minimized in the lateral plane without compromising the tissues’ integrity. Additionally, the off-line computer-aided videoframe analysis software which was used offers a digital movement-stabilization function which efficiently reduces any residual movements. An intravital microscope (Olympus BXFM, Olympus Deutschland GmbH, Hamburg, Germany) allows visual access to the vascular network within the chamber window.

Since no invasive procedures are performed, no sedation or anesthesia of the animal is necessary for the investigational timeframe.

**Fig. 4:**
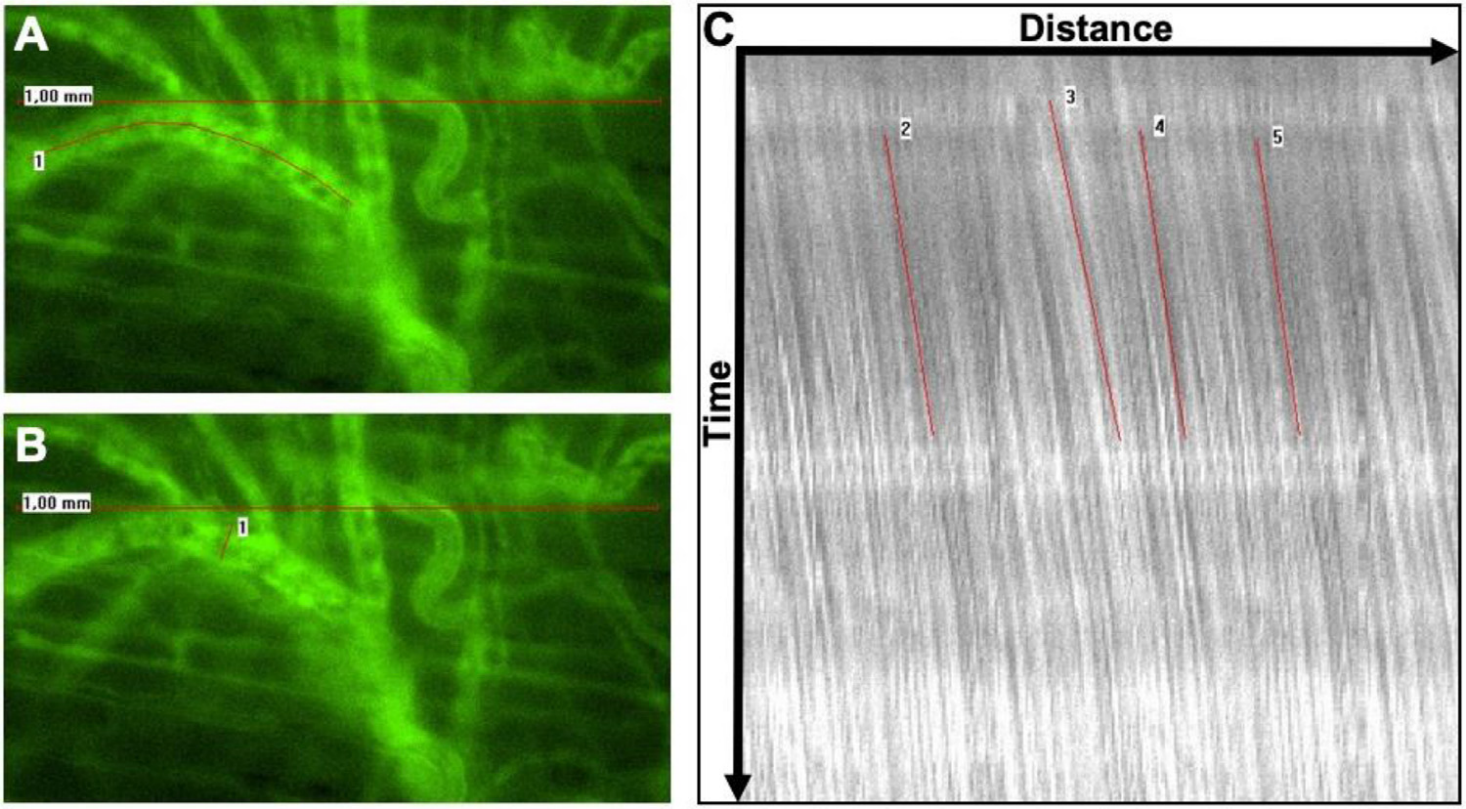
Evaluation of segmental blood flow includes labeling the course of the respective blood vessel (A, red line 1), determining the vessel diameter (B, red line 1) and carrying out Cap Image's line shift diagram function in order to calculate red blood cell velocity (C, red lines 1-5). Original data in [11], reused with permission.

In each chamber window, regions of interest (ROI) (north, east, south, west, middle) are defined. Chamber areas of insufficient quality due to non-adherence of the musculocutaneous tissue to the chamber window should be excluded from the investigation. A green filter (Excitation [Ex]:470 nm/Emission [Em]: 525 nm) is used for fluorescence imaging of the intravascular FITC-labelled dextran (Fig. 4) enhancing the contrast between erythrocytes and the fluorescent blood plasma. Furthermore, an orange filter (Ex: 545 nm; Em: 605 nm) is used for the fluorescence imaging of Rhodamine 6G accumulating in the mitochondria of white blood cells.

For each ROI, representative video sequences (∼15 s) are recorded, using the built-in microscope software cellSens Dimension (Olympus Deutschland GmbH, Hamburg, Germany) and saved as uncompressed AVI files.

Video sequences are analyzed off-line using a computer-aided videoframe analysis system (Cap-Image, Dr. Zeintl Ingenieurbüro, Dreieich, Germany) as previously described [18,19]. Within each ROI, 3,4 blood vessels are evaluated with regard to vessel diameter (d). Red blood cell velocity (V_RBC_) is determined using the line shift diagram tool. For this purpose, the course of each respective blood vessel is marked and the red blood cell flow within the vessel is automatically determined by analyzing the time-dependent brightness at each labeled point. These data are integrated into a coordinate system in which the length of the blood vessel is shown as the abscissa and the time course as the ordinate, respectively. Consequently, gaps in motion within in the line shift diagram can be identified and their velocity can be determined. In addition, each automatic evaluation is reviewed manually (Fig. 4).

By integration of the obtained parameters d and V_RBC_, the blood flow within the analyzed segment (segmental blood flow, SBF in [pl/s]) can be determined as described by Baker and Wayland [20]:

$$\text{SBF}=\left(V_{\text{RBC}}/1.6\right)x{\left(d/2\right)}^{2}x\;\pi$$

To determine the amount leukocyte rolling and adhesion, homologue rhodamine 6G enhanced video sequences are played back in slow motion (0.1 x original speed). First, the adhesive leukocytes are quantified manually using a mechanical hand counter (Roth Selection, Carl Roth GmbH + Co. KG, Karlsruhe, Germany). Each video sequence is evaluated by two independent raters, blinded with regard to the experimental group.

**Fig. 5:**
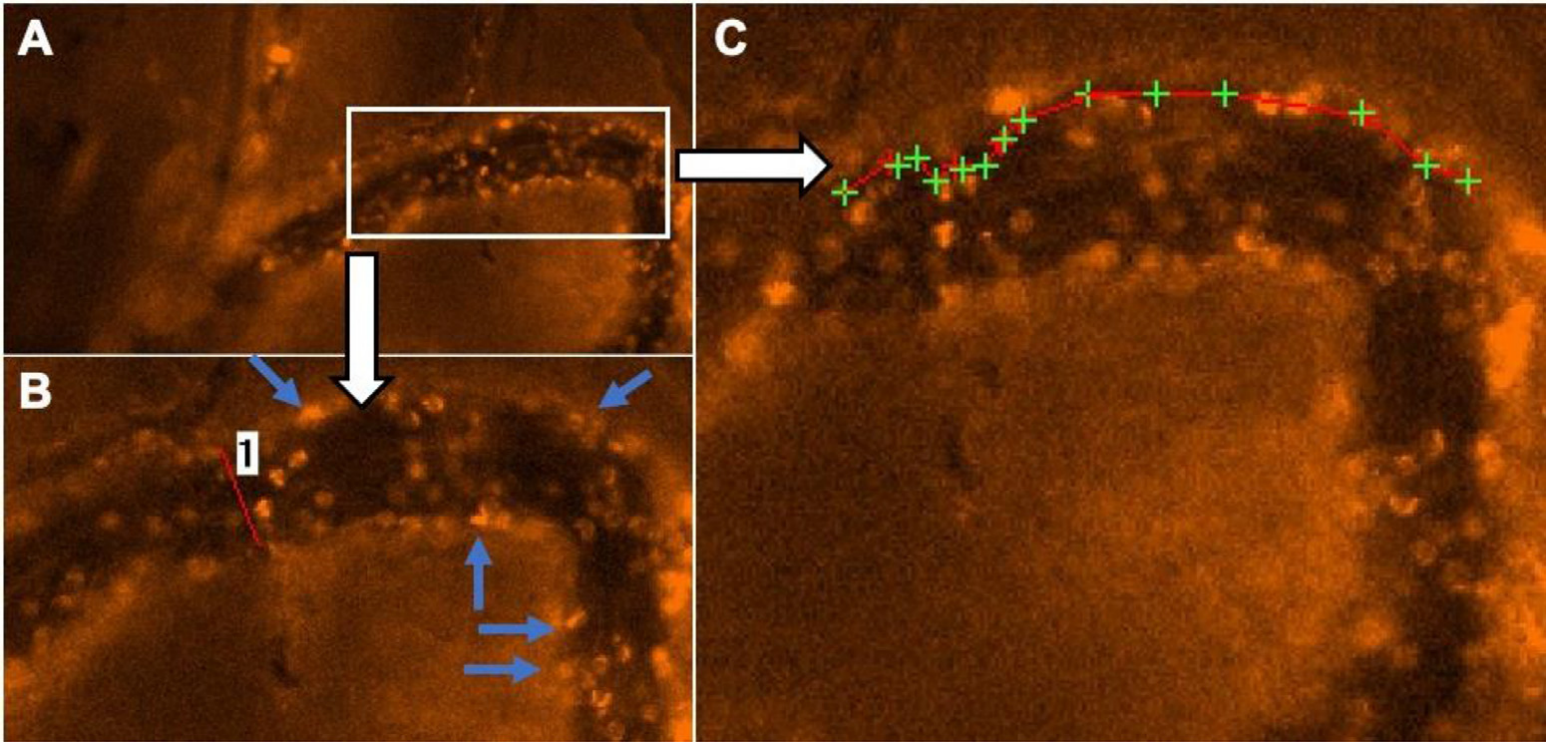
In vivo analysis of leukocyte-microvascular interactions. For evaluation of leukocyte-microvascular wall interaction rhodamine 6G enhanced intravital microscopy is used (A). Adhesive leukocytes are quantified manually using a mechanical hand counter (B, blue arrows). To quantify the amount of rolling leukocytes (i.e., decrease in flow velocity > 50%), moving leukocytes are screened visually. Decrease in velocity is subsequently verified individually by frame-to-frame analysis (C, green crosses: time-dependent leukocyte position, red line: path of rolling leukocytes). A decrease in gap between two green crosses indicates a deceleration which identifies a rolling leukocyte.

In a second step, rolling leukocytes were quantified. Leukocytes showing a reduction in velocity of >50% compared to V_RBC_ are defined as “rolling”. Using the frame-to-frame velocity analysis tool, the speed of analyzed leukocytes was determined and the respective total number quantified (Fig. 5).

## Analysis of cerebral microvascular reactivity and oxidative stress

The here described preparation method for measurement of mouse cerebral arteriole reactivity by video microscopy was established by the senior author (A.G.) [11]. Video microscopic measurement methods for arterioles of other vascular beds have previously been described by our working group [5,21]. Arterioles branching from the middle cerebral artery (MCA) were selected for measurement, since the MCA and its branches supply the largest part of the cerebral cortex and other brain regions with blood [22,23]. Moreover, most ischemic strokes occur in the territory of the MCA highlighting the pathophysiological relevance of this vascular bed [23]. While in untreated C57BL/6J mice, the main branch of the MCA is almost completely unresponsive to the endothelium-dependent vasodilator, acetylcholine, (data not shown) its small side branches display good endothelium-dependent responses which make them suitable for experimentation [11].

**Fig. 6:**
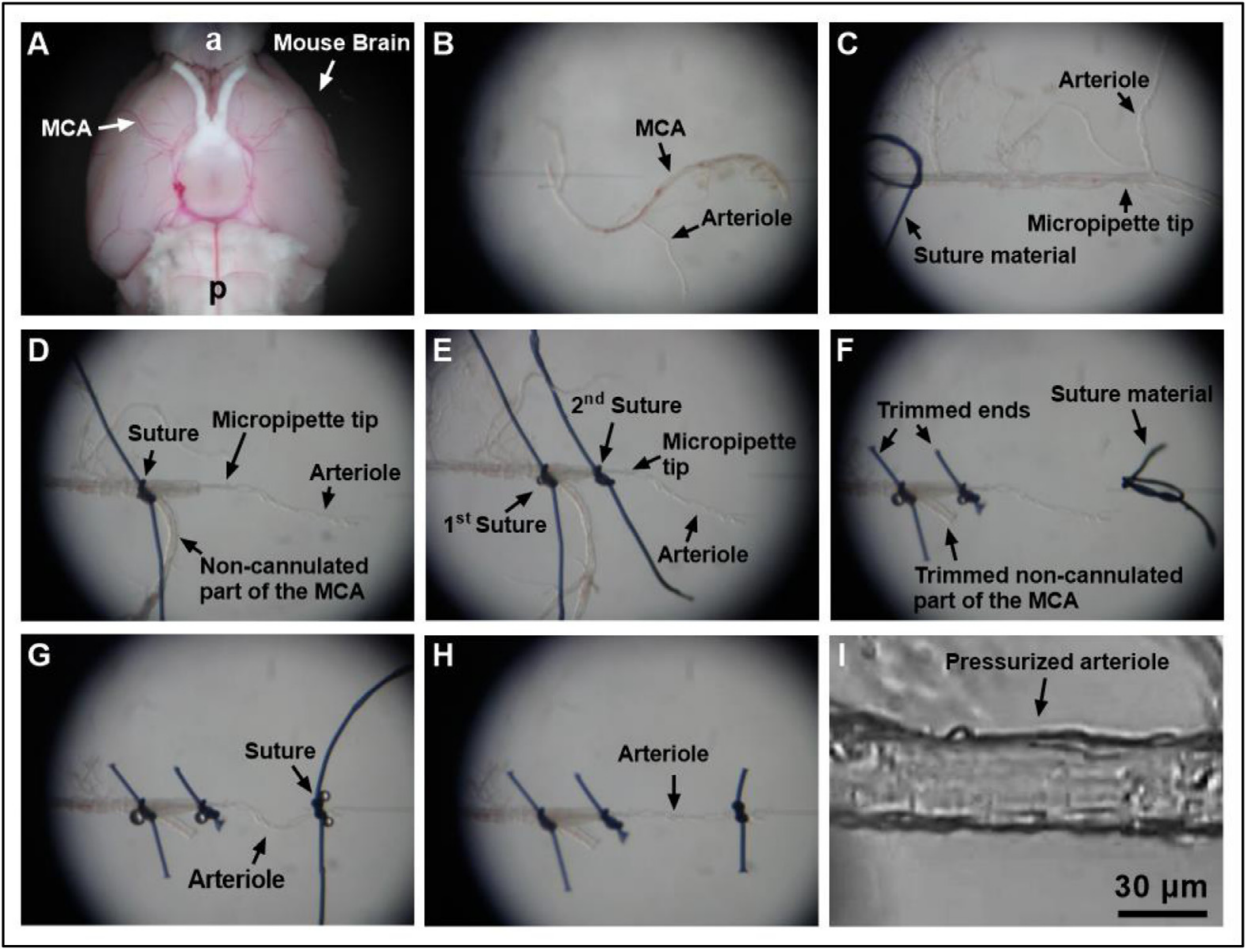
Preparation of cerebral arterioles for video microscopy. After isolation of the brain (bottom view), a vascular tree of the middle cerebral artery (MCA) is carefully isolated by using Vannas scissors and fine-point-tweezers (a, anterior; p, posterior) (A). Next, the MCA including its branches is transferred to a pressure myograph with dual micropipettes (B). The MCA is then cannulated by using two fine-point tweezers, and the micropipette tip is advanced just before the arteriole is branching. Moreover, a loop of suture (10.0 Nylon) is placed over the artery (C). The tip of the micropipette is then guided into the arteriole by gently pulling the cannulated end of the MCA further on the pipette and by pushing the free end of the MCA against the pipette tip. The arteriole is not grabbed directly at any time of the cannulation process. Once the arteriole is cannulated, the free end of the MCA is taken through the suture loop and tied to the pipette (D). To avoid leakage of fluid from side branches of the MCA and to further secure the arteriole, a second suture is put on the cannulated part of the arteriole (E). The ends of the sutures and the non-cannulated end of the MCA are then trimmed, and a loop of suture material is placed on the opposite micropipette (F). Next, the non-cannulated end of the arteriole is put through the suture loop and tied to the micropipette (G). The ends of the suture on the right micropipette are then trimmed and the micropipette tips moved apart to gently stretch the arteriole (H). Subsequently, the perfusion chamber is placed on an inverted microscope and the arteriole pressurized to 40 mmHg via the inserted micropipette and visualized by a digital camera (I). After an equilibration time of 30 min, the arteriole is ready for the measurement.

For *in vitro-*analysis, the whole mouse brain is dissected and immersed into ice cold artificial cerebrospinal fluid to ensure tissue integrity for the subsequent preparation. Next, the middle cerebral artery (MCA) as well as its arteriole branches are identified. Surrounding brain tissue is carefully removed from the vessels by means of Vannas scissors and fine-point-tweezers (Fig. 6A). Following this, the MCA and its branches are transferred to a pressure myograph with dual micropipettes (Fig. 6B). Afterwards, the MCA's lumen is cannulated with a micropipette, which is cautiously placed into one of the arteriole branches (Fig. 6C). After verification of the correct position, the surrounding vessel wall is fixed to the micropipette using dual 10.0 nylon sutures (10-0, 198001, Alcon AG, Freiburg, Switzerland, Fig. 6D,E). The end of the non-cannulated end of the MCA is then trimmed (Fig. 6F). In a final step, the opposite end of the arteriole is cannulated and fixed in the same fashion (Fig. 6G,H). For subsequent evaluations, an intravascular pressure of 40 mmHg is established under brightfield microscopic control and balanced for 30 min (Fig. 6I).

The preparation of the middle cerebral artery and its consecutive arterioles takes about 30 min. Evaluation of their individual responsiveness to various vasodilators and vasoconstrictors takes roughly another 2 h. Our preliminary experiments have shown that arterioles branching out of the middle cerebral artery may be evaluated in this context for about 6 h. Afterwards, their individual responsiveness reduces steadily.

**Fig. 7:**
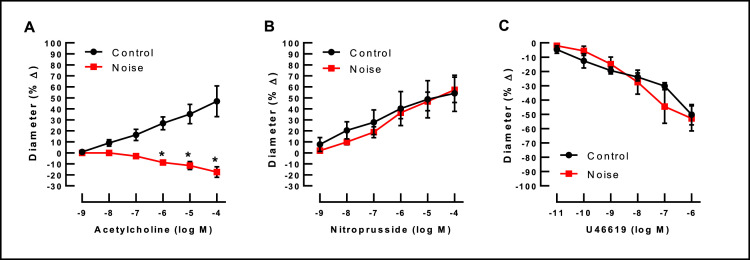
In cerebral arterioles branching from the middle cerebral artery noise markedly reduced endothelium-dependent vasodilation in response to acetylcholine. In contrast, responses to the endothelium-independent vasodilator, nitroprusside, and to the vasoconstrictor, U46619 (thromboxane A2 mimetic), remained unchanged (*n*=4 per group; 2-way ANOVA with Bonferroni's multiple comparison test). Reused from [11] with permission.

Concentration-dependent response curves of arterioles branching from the MCA are established using the endothelium-dependent vasodilator, acetylcholine (ACh, 10^−9^–10^−4^ M; Sigma-Aldrich, Taufkirchen, Germany), the endothelium-independent vasodilator, sodium nitroprusside (SNP, 10^−9^–10^−4^ M, Sigma-Aldrich), and the vasoconstricting thromboxane mimetic, U46619 (10^–11^–10^−6^ M; Cayman Chemical, Ann Arbor, MI, USA) for noise-exposed and control animals as shown in Fig. 7 [11]. For analysis of response curves to vasodilating agents, arterioles are pre-toned to about 50–70% of the original diameter by titration with U46619.

The presented cannulation method of cerebral arterioles via the main branch of the MCA is much easier, faster and less destructive than the attempt of a direct cannulation of an arteriole. Figure 6 demonstrates individual steps of the cannulation procedure for arterioles that branch from the mouse MCA in detail.

## Conclusion

The presented experimental protocol allows the visualization and quantification of effects caused by noise exposure on the activation of white blood cells microvascular dysfunction in mice [11]. Apart from noise, multiple other pathological mechanisms like infection [24], [25], [26], [27], malignant diseases [28], [29], [30], ischemia [31], metabolic disorders [32,33], pollution [34], or thermal [35,36] and chemical trauma [37] ultimately lead to inflammatory driven changes in microvascular blood flow and immune reactions. Thus, with some minor modifications, application of the experimental protocol may enable scientists to study specific effects on vascular function caused by other pathogens in the future.

## Direct submission or Co-submission

Co-submissions are papers that have been submitted alongside an original research paper accepted for publication by another Elsevier journal

Co-submission

## Declaration of Competing Interest

The authors declare that they have no known competing financial interests or personal relationships that could have appeared to influence the work reported in this paper.

## References

[1] Kupcikova Z., Fecht D., Ramakrishnan R., Clark C., Cai YS. (2021). Road traffic noise and cardiovascular disease risk factors in UK Biobank. Eur. Heart J..

[2] Munzel T., Daiber A., Steven S., Tran L.P., Ullmann E., Kossmann S. (2017). Effects of noise on vascular function, oxidative stress, and inflammation: mechanistic insight from studies in mice. Eur. Heart J..

[3] Kroller-Schon S., Daiber A., Steven S., Oelze M., Frenis K., Kalinovic S. (2018). Crucial role for Nox2 and sleep deprivation in aircraft noise-induced vascular and cerebral oxidative stress, inflammation, and gene regulation. Eur. Heart J..

[4] Steven S., Frenis K., Kalinovic S., Kvandova M., Oelze M., Helmstadter J. (2020). Exacerbation of adverse cardiovascular effects of aircraft noise in an animal model of arterial hypertension. Redox Biol..

[5] Frenis K., Helmstadter J., Ruan Y., Schramm E., Kalinovic S., Kroller-Schon S. (2021). Ablation of lysozyme M-positive cells prevents aircraft noise-induced vascular damage without improving cerebral side effects. Basic Res. Cardiol..

[6] Algire G.H., Legallais F.Y. (1949). Recent developments in the transparent-chamber technique as adapted to the mouse. J. Natl. Cancer Inst..

[7] Sandison J.C. (1924). A new method for the microscopic study of living growing tissues by the introduction of a transparent chamber in the rabbit's ear. Anat. Rec..

[8] Endrich B., Asaishi K., Gotz A., Messmer K. (1980). Technical report–a new chamber technique for microvascular studies in unanesthetized hamsters. Res. Exp. Med. (Berl.).

[9] Baatz H., Steinbauer M., Harris A.G., Krombach F. (1995). Kinetics of white blood cell staining by intravascular administration of rhodamine 6G. Int. J. Microcirc. Clin. Exp..

[10] Boulaftali Y., Lamrani L., Rouzaud M.C., Loyau S., Jandrot-Perrus M., Bouton M.C. (2012). The mouse dorsal skinfold chamber as a model for the study of thrombolysis by intravital microscopy. Thromb. Haemost..

[11] Eckrich J., Frenis K., Blanco G.R., Ruan Y., Jiang S., Bayo Jimenez M.T. (2021). Aircraft noise exposure drives the activation of white blood cells and induces microvascular dysfunction in mice. Redox Biol..

[12] Laschke M.W., Vollmar B., Menger MD. (2011). The dorsal skinfold chamber: window into the dynamic interaction of biomaterials with their surrounding host tissue. Eur. Cells Mater..

[13] Langer S., Beescho C., Ring A., Dorfmann O., Steinau H.U., Spindler N. (2016). A new in vivo model using a dorsal skinfold chamber to investigate microcirculation and angiogenesis in diabetic wounds. GMS Interdiscip. Plast. Reconstr. Surg. DGPW.

[14] Mussawy H., Viezens L., Hauenherm G., Schroeder M., Schaefer C. (2017). In vivo functional and morphological characterization of bone and striated muscle microcirculation in NSG mice. PLoS One.

[15] Lehr H.A., Leunig M., Menger M.D., Nolte D., Messmer K. (1993). Dorsal skinfold chamber technique for intravital microscopy in nude mice. Am. J. Pathol..

[16] Reichel C.A., Hessenauer M.E., Pflieger K., Rehberg M., Kanse S.M., Zahler S. (2015). Components of the plasminogen activation system promote engraftment of porous polyethylene biomaterial via common and distinct effects. PLoS One.

[17] Munzel T., Schmidt F.P., Steven S., Herzog J., Daiber A., Sorensen M. (2018). Environmental Noise and the Cardiovascular System. J. Am. Coll. Cardiol..

[18] Zeintl H., Sack F.U., Intaglietta M., Messmer K. (1989). Computer assisted leukocyte adhesion measurement in intravital microscopy. Int. J. Microcirc. Clin. Exp..

[19] Klyscz T., Junger M., Jung F., Zeintl H. (1997). [Cap image-a new kind of computer-assisted video image analysis system for dynamic capillary microscopy]. Biomed. Tech. (Berl.).

[20] Baker M., Wayland H. (1974). On-line volume flow rate and velocity profile measurement for blood in microvessels. Microvasc. Res..

[21] Gericke A., Goloborodko E., Pfeiffer N., Manicam C. (2018). Preparation steps for measurement of reactivity in mouse retinal arterioles *ex vivo*. J. Vis. Exp..

[22] Carmichael S.T. (2005). Rodent models of focal stroke: size, mechanism, and purpose. NeuroRx.

[23] Navarro-Orozco D., Sanchez-Manso J.C. (2021). Neuroanatomy, middle cerebral artery. StatPearls.

[24] Cryer H.M., Garrison R.N., Kaebnick H.W., Harris P.D., Flint LM. (1987). Skeletal microcirculatory responses to hyperdynamic Escherichia coli sepsis in unanesthetized rats. Arch. Surg..

[25] De Backer D., Creteur J., Preiser J.C., Dubois M.J., Vincent JL. (2002). Microvascular blood flow is altered in patients with sepsis. Am. J. Respir. Crit. Care Med..

[26] Piper R.D., Pitt-Hyde M., Li F., Sibbald W.J., Potter R.F. (1996). Microcirculatory changes in rat skeletal muscle in sepsis. Am. J. Respir. Crit. Care Med..

[27] Pober J.S., Sessa W.C. (2014). Inflammation and the blood microvascular system. Cold Spring Harb. Perspect. Biol..

[28] Brown E.B., Campbell R.B., Tsuzuki Y., Xu L., Carmeliet P., Fukumura D. (2001). In vivo measurement of gene expression, angiogenesis and physiological function in tumors using multiphoton laser scanning microscopy. Nat. Med..

[29] Dubina M.V., Petrishchev N.N., Anisimov V.N. (1999). Microvascular endothelium dysfunction during growth of transplanted lymphosarcoma and glioma in rats. J. Exp. Clin. Cancer Res..

[30] Schaefer C., Krause M., Fuhrhop I., Schroeder M., Algenstaedt P., Fiedler W. (2008). Time-course-dependent microvascular alterations in a model of myeloid leukemia in vivo. Leukemia.

[31] Uz Z., Ince C., Shen L., Ergin B., van Gulik T.M. (2021). Real-time observation of microcirculatory leukocytes in patients undergoing major liver resection. Sci. Rep..

[32] Algenstaedt P., Schaefer C., Biermann T., Hamann A., Schwarzloh B., Greten H. (2003). Microvascular alterations in diabetic mice correlate with level of hyperglycemia. Diabetes.

[33] Bono P., Jalkanen S., Salmi M. (1999). Mouse vascular adhesion protein 1 is a sialoglycoprotein with enzymatic activity and is induced in diabetic insulitis. Am. J. Pathol..

[34] Suwannasual U., Lucero J., McDonald J.D., Lund AK. (2018). Exposure to traffic-generated air pollutants mediates alterations in brain microvascular integrity in wildtype mice on a high-fat diet. Environ. Res..

[35] Boykin J.V., Eriksson E., Pittman RN. (1980). In vivo microcirculation of a scald burn and the progression of postburn dermal ischemia. Plast. Reconstr. Surg..

[36] Goertz O., Vogelpohl J., Jettkant B., Daigeler A., Steinau H.U., Steinstraesser L. (2009). Burn model for in vivo investigations of microcirculatory changes. Eplasty.

[37] Goertz O., Popp A., Kolbenschlag J., Vogelpohl J., Daigeler A., Ring A. (2013). Intravital pathophysiological comparison of acid- and alkali-burn injuries in a murine model. J. Surg. Res..

